# Waist circumference and a body shape index and prostate cancer risk and mortality

**DOI:** 10.1002/cam4.3827

**Published:** 2021-03-12

**Authors:** Sylvia H. J. Jochems, Angela M. Wood, Christel Häggström, Marju Orho‐Melander, Pär Stattin, Tanja Stocks

**Affiliations:** ^1^ Department of Clinical Sciences Lund Lund University Lund Sweden; ^2^ Department of Public Health and Primary Care University of Cambridge Cambridge UK; ^3^ Department of Public Health and Clinical Medicine Umeå University Umeå Sweden; ^4^ Department of Surgical Sciences Uppsala University Uppsala Sweden; ^5^ Department of Clinical Sciences Malmö Lund University Lund Sweden

**Keywords:** cancer risk factors, epidemiology, prognosis, prostate cancer

## Abstract

We recently found a negative association between body mass index (BMI) and the risk of localised prostate cancer (PCa), no association with advanced PCa, and a positive association with PCa‐specific mortality. In a 15% subpopulation of that study, we here investigated the measures of abdominal adiposity including waist circumference (WC) and A Body Shape Index (ABSI) in relation to PCa risk and mortality. We used data from 58,457 men from four Swedish cohorts to assess WC and ABSI in relation to PCa risk according to cancer risk category, including localised asymptomatic and symptomatic PCa and advanced PCa, and PCa‐specific mortality. Cox regression models were used to calculate hazard ratios (HRs) and 95% confidence intervals (CIs). During, on average, 10 years of follow‐up, 3290 men were diagnosed with PCa and 387 died of PCa. WC was negatively associated with the risk of total PCa (HR per 10 cm, 0.95; 95% CI 0.92–0.99), localised PCa (HR per 10 cm, 0.93, 95% CI 0.88–0.96) and localised asymptomatic PCa cases detected through a prostate‐specific antigen (PSA) test (HR per 10 cm, 0.87, 95% CI 0.81–0.94). WC was not associated with the risk of advanced PCa (HR per 10 cm, 1.02, 95% CI 0.93–1.14) or with PCa‐specific mortality (HR per 10 cm, 1.04, 95% CI 0.92–1.19). ABSI showed no associations with the risk of PCa or PCa‐specific mortality. While the negative association between WC and the risk of localised PCa was partially driven by PSA‐detected PCa cases, no association was found between abdominal adiposity and clinically manifest PCa in our population.

## Graphical abstract

This study provides a more complete picture of the association between abdominal adiposity and the risk of prostate cancer and the mortality associated with it. The findings support a prevailing, yet poorly tested, hypothesis that the negative association between waist circumference and the risk of localised prostate cancer may be partly driven by normal‐weight men undergoing prostate cancer screening more often than obese men. The negative association between waist circumference and the risk of localised prostate cancer is partially driven by asymptomatic prostate cancer’s detected through a prostate‐specific antigen test.

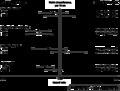

## 1 INTRODUCTION

Obesity is a rapidly growing public health concern and accurate measures of body fatness are needed to clarify its role in the incidence and prognosis of prostate cancer (PCa).^1^ In most previous observational studies, body mass index (BMI), a marker of general obesity, has been found to be negatively associated with the risk of localised PCa, and positively associated with advanced PCa risk.^2^, ^3^, ^4^ However, in our recent study including 32,871 incident PCa cases^5^ and another large pooled analysis by Genkinger et al,^6^ only little evidence was found for an association between BMI and the risk of more advanced PCa. Also, findings from Mendelian randomisation studies suggest no strong evidence of a causal effect of BMI on PCa.^7^, ^8^ In addition to BMI, anthropometric measures of abdominal adiposity including waist circumference (WC) are also suggested to influence PCa incidence. Although less consistent, studies investigating WC and PCa risk also found mostly null or negative associations with localised PCa risk but null or positive associations with advanced PCa risk.^3^, ^4^, ^6^, ^9^, ^10^, ^11^, ^12^ A body shape index (ABSI), a measure based on WC, weight and height and independent from BMI, showed no association with the risk of PCa.^10^ The various findings for anthropometric measures across PCa risk categories, and the different prognosis of PCa even within specific subgroups,^13^ highlight the diversity of PCa and the need for further investigation by use of detailed clinical characteristics of the PCa.

The differential associations of PCa by disease severity have been hypothesised to be attributed to clinical characteristics of men with obesity that may influence the detection of PCa, such as a lower serum prostate‐specific antigen (PSA) concentration,^14^, ^15^, ^16^ and a larger prostate gland compared to normal weight men, which may lower the detection through biopsy.^17^ Moreover, more active screening behaviour in normal weight men compared to men with obesity may be involved. In our aforementioned study, we found that the negative association between BMI and the risk of localised PCa was partially driven by PCa cases detected through asymptomatic testing,^5^ which supports a role for detection bias in the obesity–PCa association. To the best of our knowledge, no other prospective study has tested the prevailing hypothesis that the negative association between adiposity and the risk of localised PCa may be partly driven by localised PCa cases detected through PSA‐testing.

Despite the lack of an association between BMI and more advanced PCa in our previous study,^5^ we, and a large number of other studies, found a positive association between BMI and the risk of PCa‐specific mortality.^6^, ^18^, ^19^, ^20^, ^21^, ^22^ Only a few studies reported findings for WC and PCa‐specific mortality, which varied from no association^23^, ^24^ to a positive association.^3^, ^6^ Although ABSI has been found to be positively associated with all‐cause mortality,^25^, ^26^, ^27^ its role in cancer‐specific mortality has yet to be confirmed.^26^

We investigated the associations between abdominal adiposity measures including WC and ABSI and PCa risk, by cancer risk category and the reason for PCa detection (asymptomatic or symptomatic), and PCa‐specific mortality, in a subset of men of our previous study of body size and PCa.

## 2 METHODS

### 2.1 Study population

Out of the five cohorts included in our original investigation of general obesity and PCa,^5^ the Swedish Construction Workers Cohort [CWC] (‘Bygghälsan’),^28^ the Västerbotten Intervention Programme [VIP],^29^, ^30^ the Northern Sweden Monica [MONICA] study,^29^, ^31^ the Malmö Diet and Cancer Study [MDCS]^32^ and the Malmö Preventive Project [MPP],^33^ WC had been measured in all but the largest cohort, the CWC, and only during later years in the VIP (2003 onwards), and during a re‐examination period (2002–2006) in the MPP. In all cohorts, height, weight and WC were measured at a health examination by trained staff.

### 2.2 Selection criteria

After excluding examinations: from duplicate cohorts in men who had participated in more than one cohort, performed before 18 years of age, with a prevalent cancer (excluding non‐melanoma skin cancer), with mismatching dates, and with missings for WC, height or weight, a total of 58,457 men were included in the study, comprising approximately 15% of the population in our previous study.^5^

### 2.3 Follow‐up

The unique personal identification number of all inhabitants in Sweden was used to follow‐up cohort participants in national registers until 31 December 2016. The Swedish Cancer Register^34^ was used for the identification of diagnoses of PCa cases (International Classification of Diseases, version seven [ICD‐7] codes 177 or ICD‐10 C61) and other cancers. To obtain information on the primary underlying cause of death, the Swedish Cause of Death Register^35^ was used, which has a concordance of 86% with medical records for PCa‐related deaths. Individuals were also linked to other nationwide registers including the Total Population Register for information on migration, the Longitudinal Integration Database for Health Insurance and Labour Market Studies (LISA) for information on socioeconomic factors and country of birth, and the Patient Register for information on in‐patient care which we used for the Charlson comorbidity index.^36^

The National Prostate Cancer Register (NPCR) of Sweden became nationwide in 1998 has captured 99% of all cases of PCa, and was used to classify PCa cases into five risk groups according to clinical information at PCa diagnosis: localised low‐risk (T1‐2, Gleason score 2–6 and PSA < 10 ng/ml), localised intermediate risk (T1‐2, Gleason score 7 and/or PSA 10–<20 ng/ml), localised high risk (T3 and/or Gleason score 8–10 and/or PSA 20 to <50 ng/ml), regionally metastatic/locally advanced (T4 and/or N1 and/or PSA 50 to <100 ng/ml in the absence of distant metastases [M0 or Mx]) or distant metastases (M1 and/or PSA ≥ 100 ng/ml).^37^, ^38^ Localised PCa was further investigated by the main reason for detecting the disease, which was recorded as of the year 2000 and was categorised into asymptomatic detection (through a PSA‐test) and symptomatic detection including lower urinary tract symptoms (LUTS) or any other symptoms.

### 2.4 Statistical analysis

Cox regression models with attained age as the time scale were used to calculate hazard ratios (HRs) and 95% confidence intervals (95% CIs) for PCa incidence according to cancer risk category and detection mode of localised PCa, and for PCa‐specific mortality, by levels of WC (per 10 cm increase and categories <94, 94–102, >102 cm), ABSI (per standard deviation increase) and BMI (per 5 kg/m^2^ increase) in the full population. Person‐years for each individual were counted from the date of study enrolment until the date of a first PCa diagnosis, another cancer diagnosis (excluding non‐melanoma skin cancer), emigration, death or end of follow‐up (31 December 2016). Follow‐up in the analysis of PCa risk category and by detection mode started on 1 January 1998 or 2000, respectively, or at study enrolment, whichever occurred last. Models were stratified for cohort and birth period (<1935, 1935–1939, 1940–1944, 1945–1949 and ≥1950), and adjustment for the a priori chosen variables: age at study enrolment (continuous), smoking status at study enrolment (never smoker, ex‐smoker, current smoker), healthcare region (North, Uppsala‐Örebro, Stockholm, West, South‐East, South), country of birth (born in Sweden with both parents born in Sweden, born in Sweden with one parent born in Sweden, born in Sweden with both parents born abroad, born abroad, missing) and highest education at study enrolment (pre‐upper secondary school <9 years, pre‐upper secondary school 9 years, max. 2 years upper secondary school, 3 years upper secondary school, post‐upper secondary school <3 years, post‐upper secondary school ≥3 years including university, missing). We additionally adjusted the analysis of WC for height (continuous).

We tested the interaction between cohort and WC and ABSI, respectively, in relation to PCa risk, using the likelihood ratio test. We found no interaction between cohort and WC (*p* = 0.7921) or between cohort and ABSI (*p* = 0.4984), which supported the pooling of the cohorts into one analysis. The assumption of proportional hazards was examined for the relationship of scaled Schoenfeld residuals with time and appeared to be violated for both age at study enrolment and birth period. However, as including age as a stratum did not alter the estimates, we only stratified for birth period and cohort (to control for any differences in PCa hazards over time between the cohorts). The heterogeneity between the PCa risk categories was calculated using the duplication method for Cox regression as described by Lunn and McNeil.^39^

In the analysis of PCa cases only, Cox regression models with time since diagnosis as the time scale were used to calculate HRs and 95% CIs for PCa‐specific mortality by levels of WC, ABSI and BMI. Adjustments were the same as in the full population analysis for smoking status, healthcare region, country of birth and height, and additional adjustments were made for age at PCa diagnosis (continuous), highest education closest to diagnosis, income closest to diagnosis (<158, 158–193, 193–230, ≥230 kSEK/year, missing), source of income closest to diagnosis (work, studies, care of child/family, sick, unemployed, early retirement, social benefits, labour market policy activity, pensioner, no income, missing), civil status closest to diagnosis (unmarried, married, divorced, widower, missing), comorbidity according to the Charlson comorbidity index (none, mild, severe), primary treatment (conservative, curative, non‐curative, missing) and PCa risk category (in the analysis for total PCa). We analysed WC as a continuous variable (per 10 cm) and in categories (<94 cm, 94–102 cm, >102 cm) using the World Health Organisation's cut‐offs for WC in Caucasian men.^40^ ABSI was calculated with the formula^27^: 1000 × WC × weight −2/3 × height 5/6, and analysed per standard deviation (SD) increase. In addition, BMI per 5 kg/m^2^ was analysed to explore whether any differences in associations with BMI compared to our previous analysis could be due to a smaller study sample.

A total of 13,912 men had at least one repeated measurement of WC. Therefore, we calculated the regression dilution ratio (RDR) of WC, ABSI and BMI, in order to account for the short‐ and long‐term intra‐individual variation including random measurement errors.^41^ The RDR was 0.85 for WC and 0.95 for ABSI in the full population, and 0.91 for WC and 0.94 for ABSI among PCa cases only. The RDR for BMI was 0.90 for both the full population and the cases. All HRs were RDR‐corrected using the formula: HR_corrected _= exp(log[HR_original_]/RDR).

Statistical tests were two‐sided and data were analysed with STATA release 13 (College Station, StataCorp LP).

## 3 RESULTS

Baseline characteristics of the 58,457 men according to WC are presented in Table 1, and according to cohort in Table S1 in the Appendix 1. During on average 10.7 years (SD 6.3) of follow‐up, 3290 men were diagnosed with PCa, of which 387 died from the disease. For these 3290 men, clinical characteristics are presented in Table 2. A total of 2470 men had sufficient information in the NPCR for cancer risk categorisation and 2340 men also on the main reason for PCa detection. The median time of follow‐up among PCa cases from the date of study enrolment until PCa diagnosis was 8.5 years and the median time from PCa diagnosis to PCa‐specific mortality was 4.1 years.

**TABLE 1:** Baseline characteristics of the 58,457 men in the study, in total and according to waist circumference

Baseline characteristic		Per waist circumference, cm		
	Total	<94	94–102	>102
	(*n* = 58,457)	(*n* = 24,379)	(*n* = 17,473)	(*n* = 16,605)
Cohort (year of baseline examination), *n* (%)				
Västerbotten Intervention Programme (2003–2016)	37,396 (64)	14,782 (60)	11,349 (65)	11,265 (68)
Northern Sweden Monica Study (1986–2014)	4000 (7)	1967 (8)	1139 (6)	894 (5)
Malmö Diet and Cancer Study (1991–1996)	11,615 (20)	6014 (25)	3235 (19)	2366 (14)
Malmö Preventive Project (2002–2006)	5446 (9)	1616 (7)	1750 (10)	2080 (13)
Age at study enrolment, years				
Mean (SD)	53.0 (10.2)	51.7 (10.5)	53.7 (9.9)	54.1 (9.9)
Height, cm				
Mean (SD)	178.1 (6.8)	177.1 (6.7)	178.4 (6.7)	179.2 (6.8)
Weight, kg				
Mean (SD)	85.4 (13.7)	75.2 (7.8)	85.7 (7.1)	100.0 (12.4)
Body mass index, kg/m^2^				
Mean (SD)	26.9 (3.9)	24.0 (2.1)	26.9 (1.9)	31.1 (3.5)
Categories, *n* (%)				
<25	19,352 (33)	16,588 (67)	2620 (15)	144 (1)
25–30	28,510 (49)	7737 (32)	13,846 (79)	6927 (42)
>30	10,595 (18)	54 (1)	1007 (6)	9534 (57)
Smoking status, *n* (%)				
Never smoker	32,069 (55)	14,287 (59)	9518 (54)	8264 (50)
Ex‐smoker	15,854 (27)	5426 (22)	4987 (29)	5441 (33)
Current smoker	8871 (15)	4011 (16)	2472 (14)	2388 (14)
Missing	1663 (3)	655 (3)	496 (3)	512 (3)
Highest education, *n* (%)^a^				
Pre‐upper secondary school <9 years	7209 (12)	2828 (12)	2214 (13)	2167 (13)
Pre‐upper secondary school 9 years	4566 (8)	1573 (6)	1397 (8)	1596 (10)
Max 2 years upper secondary school	19,961 (34)	7694 (32)	6047 (35)	6220 (37)
3 years upper secondary school	9563 (16)	4102 (17)	2802 (16)	2659 (16)
Post‐upper secondary school <3 years	7568 (13)	3288 (13)	2301 (13)	1979 (12)
Post‐upper secondary school ≥3 years	9343 (16)	4785 (20)	2656 (15)	1902 (11)
Missing	247 (1)	109 (1)	56 (1)	82 (1)
Country of birth, *n* (%)				
Born in Sweden and both parents born in Sweden	51,116 (87)	21,253 (87)	15,311 (88)	14,552 (88)
Other	7341 (13)	3126 (13)	2162 (12)	2053 (12)

Abbreviation: SD, standard deviation.

^a^ Determined by the Swedish Longitudinal integration database for health insurance and labour market studies.

**TABLE 2:** Clinical characteristics of the 3290 incident prostate cancer cases in the study, in total and according to cohort

Clinical characteristic	Total	Västerbotten Intervention Programme (VIP)	Northern Sweden Monica Study (MONICA)	Malmö Diet and Cancer Study (MDCS)	Malmö Preventive Project (MPP)
	(*n* = 3290)	(*n* = 947)	(*n* = 257)	(*n* = 1623)	(*n* = 463)
Follow‐up time from study enrolment to diagnosis, years					
Mean (SD)	9.5 (6.2)	6.1 (3.4)	13.9 (7.9)	11.8 (6.2)	5.8 (3.5)
Year of diagnosis					
Mean (SD)	2009	2013	2008	2006	2010
Follow‐up time since diagnosis, years					
Mean (SD)	6.5 (4.7)	4.3 (2.9)	5.7 (4.4)	8.4 (5.4)	5.6 (3.5)
Age at diagnosis, years					
Mean (SD)	69.3 (7.4)	63.4 (5.4)	71.9 (7.6)	71.4 (6.7)	72.8 (6.5)
Charlson comorbidity index, *n* (%)^a^					
0 (no comorbidity)	2564 (78)	833 (88)	194 (75)	1182 (73)	355 (77)
1 (mild comorbidity)	296 (9)	55 (6)	22 (9)	169 (10)	50 (11)
≥2 (severe comorbidity)	220 (7)	27 (3)	25 (10)	140 (9)	28 (6)
Missing	210 (6)	32 (3)	16 (6)	132 (8)	30 (6)
Detection mode of the prostate cancer^b^					
Asymptomatic (through a PSA‐test)	1316 (40)	506 (54)	76 (31)	531 (35)	203 (45)
Lower urinary tract symptoms	844 (26)	267 (29)	80 (32)	331 (22)	166 (37)
Other symptoms	706 (21)	148 (16)	61 (24)	422 (27)	75 (17)
Missing	424 (13)	6 (1)	32 (13)	244 (16)	7 (1)
Local clinical tumour stage, *n* (%)					
T0	38 (1)	2 (<1)	1 (<1)	30 (2)	5 (1)
T1a, b	151 (4)	15 (2)	14 (5)	91 (5)	31 (7)
T1c	1439 (44)	584 (61)	89 (35)	586 (36)	180 (39)
T1 unspecified	30 (1)	6 (1)	4 (2)	13 (1)	7 (1)
T2	919 (28)	245 (26)	77 (30)	474 (29)	123 (27)
T3, 4	578 (18)	75 (8)	64 (25)	334 (21)	105 (23)
Missing	135 (4)	20 (2)	8 (3)	95 (6)	12 (3)
Lymph node metastasis, *n* (%)					
N0, no lymph node metastasis	582 (18)	171 (18)	31 (12)	317 (20)	63 (14)
N1, lymph node metastasis	113 (3)	44 (5)	9 (4)	39 (2)	21 (4)
Nx, no lymph node extirpation performed^c^	2453 (75)	712 (75)	209 (81)	1165 (72)	367 (79)
Missing	142 (4)	20 (2)	8 (3)	102 (6)	12 (3)
Bone metastasis, *n* (%)					
M0, no bone metastasis	1939 (59)	729 (77)	130 (51)	834 (51)	246 (53)
M1, bone metastasis	258 (8)	53 (6)	37 (14)	120 (8)	48 (10)
Mx, no bone scan performed^c^	951 (29)	145 (15)	82 (32)	567 (35)	157 (34)
Missing	142 (4)	20 (2)	8 (3)	102 (6)	12 (3)
Tumour differentiation, *n* (%)^d^					
Low grade	214 (6)	0 (0)	17 (7)	192 (12)	5 (1)
Intermediate grade	450 (14)	1 (<1)	25 (10)	398 (25)	26 (6)
High grade	220 (7)	2 (<1)	9 (3)	196 (12)	13 (3)
Gx^c^	51 (2)	0 (0)	1 (<1)	50 (3)	0 (0)
Missing	2355 (71)	944 (99)	205 (80)	787 (48)	419 (90)
Cancer risk category, *n* (%)^e^					
Localised low risk	905 (28)	350 (37)	37 (14)	423 (26)	95 (21)
Localised intermediate risk	954 (29)	319 (34)	82 (32)	416 (26)	137 (30)
Localised high risk	703 (21)	147 (16)	52 (20)	392 (24)	112 (24)
Regionally metastatic/locally advanced	189 (6)	39 (4)	22 (9)	98 (6)	30 (6)
Distant metastases	329 (10)	60 (6)	48 (19)	162 (10)	59 (13)
Missing	210 (6)	32 (3)	16 (6)	132 (8)	30 (6)
Primary treatment, *n* (%)^f^					
Conservative	863 (26)	265 (28)	69 (27)	392 (24)	137 (30)
Curative	1451 (44)	561 (59)	73 (29)	656 (41)	161 (35)
Non‐curative	766 (23)	61 (7)	101 (39)	458 (28)	146 (31)
Dead before treatment decision	7 (1)	2 (<1)	0 (0)	3 (<1)	2 (<1)
Missing	203 (6)	58 (6)	14 (5)	114 (7)	17 (4)

Abbreviations: PSA, prostate‐specific antigen; SD, standard deviation.

^a^ Based on discharge diagnoses in the Swedish Patient Register.

^b^ This information was recorded as of the year 2000, that is, 2 years after the National Prostate Cancer Register of Sweden became nationwide.

^c^ Nx, Mx and Gx imply that these were never measured, and the reason for missing data is unknown.

^d^ Classified according to Gleason grading or WHO grade into low grade (Gleason score 2–6 or WHO grade 1), intermediate grade (Gleason score 7 or WHO grade 2) or high grade (Gleason score ≥8 or WHO grade 3).

^e^ Localised low risk, T1‐2, Gleason score 2–6 and PSA < 10 ng/ml; localised intermediate risk, T1‐2, Gleason score 7 and/or PSA 10 to <20 ng/ml; localised high risk, T3 and/or Gleason score 8–10 and/or PSA 20 to <50 ng/ml; regionally metastatic/locally advanced, T4 and/or N1 and/or PSA 50 to <100 ng/ml in the absence of distant metastases; distant metastases, M1 and/or PSA ≥ 100 ng/ml.

^f^ Conservative treatment includes watchful waiting and active surveillance; curative treatment includes radical prostatectomy and radiotherapy; non‐curative treatment includes all androgen deprivation therapies (orchiectomy, GnRH agonists and antagonists) and antiandrogens.

**FIGURE 1:**
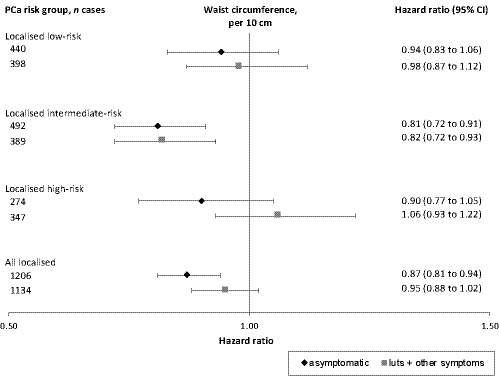
Hazard ratios (95% confidence intervals [CIs]) for incident localised prostate cancer, assigned a cancer risk category and by mode of detection, according to waist circumference per 10 cm. Prostate cancer risk categories were categorised into localised low‐risk = T1‐2, Gleason score 2‐6 and PSA <10 ng/ml; localised intermediate‐risk = T1‐2, Gleason score 7 and/or PSA 10 to <20 ng/ml; and localised high‐risk = T3 and/or Gleason score 8‐10 and/or PSA 20 to <50 ng/ml. Hazard ratios (95% CI) were calculated by Cox regression with attained age as time scale, stratified on cohort and birth decade, and adjusted for age at study entry, height, smoking status, healthcare region, country of birth, and highest education. Hazard ratios of waist circumference were corrected for a regression dilution ratio (RDR) of 0.85. LUTS = lower urinary tract symptoms; PCa = prostate cancer

WC was negatively associated with the risk of total PCa (HR per 10 cm, 0.95; 95% CI 0.92–0.99) and all localised PCa (HR per 10 cm, 0.93, 95% CI 0.88–0.96), but was not associated with more advanced PCa (*p* for heterogeneity between risk groups = 0.09) (Table 3). For all localised PCa cases, the negative association with WC was the strongest for asymptomatic PCa cases (HR per 10 cm, 0.87; 95% CI 0.81–0.94), and there were no associations with localised PCa cases detected by LUTS or any other symptoms (HR per 10 cm, 0.95; 95% CI 0.88–1.02); however, confidence intervals were largely overlapping between groups (Figure 1). WC was not associated with the risk of dying of PCa in the full population (HR per 10 cm, 1.07, 95% CI 0.95–1.20) or among PCa cases (HR per 10 cm, 1.04, 95% CI 0.92–1.19). WC was positively associated with all‐cause mortality (HR per 10 cm, 1.08, 95% CI 1.01–1.06) among PCa cases.

**TABLE 3:** Hazard ratio's (95% confidence interval) of incident prostate cancers according to cancer risk category and prostate cancer‐specific mortality, by level of waist circumference and body mass index, in the full population and among cases only

Prostate cancer outcome	Waist circumference, cm				ABSI	BMI^f^, kg/m^2^	Number of individuals in each analysis
	<94	94–102	>102	Per 10	Per SD	Per 5	
	ref	HR (95% CI)	HR (95% CI)	HR (95% CI)	HR (95% CI)	HR (95% CI)	
All localised	1.00	0.94 (0.85–1.05)	0.77 (0.68–0.87)	0.93 (0.88–0.96)	0.97 (0.93–1.02)	0.90 (0.85–0.97)	Full population as of 1.1.1998 with NPCR information, *n* = 57,566^b^
*n* cases	1126	781	563	2470	2470	2470	
Localised low risk^a^	1.00	1.17 (0.96–1.39)	0.79 (0.64–0.99)	0.95 (0.88–1.04)	0.98 (0.90–1.05)	0.98 (0.88–1.09)	
*n* cases	371	313	189	873	873	873	
Localised intermediate risk^a^	1.00	0.84 (0.70–1.00)	0.66 (0.53–0.80)	0.85 (0.79–0.92)	0.93 (0.86–1.01)	0.81 (0.73–0.90)	
*n* cases	450	282	198	930	930	930	
Localised high risk^a^	1.00	0.83 (0.67–1.04)	0.94 (0.75–1.18)	0.99 (0.91–1.07)	1.05 (0.96–1.14)	0.94 (0.83–1.08)	
*n* cases	305	186	176	667	667	667	
All advanced	1.00	1.04 (0.81–1.34)	1.05 (0.79–1.37)	1.02 (0.93–1.14)	0.96 (0.86–1.06)	1.04 (0.90–1.21)	
*n* cases	204	154	125	483	483	483	
Regionally metastatic/locally advanced^a^	1.00	0.98 (0.64–1.50)	1.25 (0.80–1.94)	1.05 (0.88–1.24)	0.92 (0.72–1.01)	1.17 (0.91–1.49)	
*n* cases	79	45	53	177	177	177	
Distant metastases^a^	1.00	1.07 (0.79–1.46)	0.94 (0.66–1.34)	1.01 (0.89–1.15)	1.01 (0.89–1.14)	0.98 (0.81–1.19)	
*n* cases	125	109	72	306	306	306	
*p* for heterogeneity between the five prostate cancer risk categories^c^		0.94	0.08	0.09	0.46	0.22	
All incident prostate cancers^d^	1.00	0.95 (0.86–1.05)	0.84 (0.75–0.93)	0.95 (0.92–0.99)	0.97 (0.93–1.00)	0.94 (0.89–0.99)	Full population, *n* = 58,457^b^
*n* cases	1487	1029	774	3290	3290	3290	
Prostate cancer‐specific mortality	1.00	1.30 (0.99–1.70)	1.23 (0.89–1.69)	1.07 (0.95–1.20)	0.99 (0.90–1.09)	1.13 (0.97–1.35)	
*n* cases	157	136	94	387	387	387	
All‐cause mortality	1.00	1.06 (1.00–1.11)	1.40 (1.33–1.48)	1.14 (1.12–1.16)	1.20 (1.18–1.22)	1.10 (1.06–1.14)	
*n* cases	3148	2713	2482	8343	8343	8343	
Prostate cancer‐specific mortality	1.00	1.19 (0.90–1.57)	1.20 (0.86–1.66)	1.04 (0.92–1.19)	0.96 (0.85–1.08)	1.17 (0.98–1.42)	PCa cases with NPCR information, *n* = 3080^e^
*n* cases	146	128	86	360	360	360	
All‐cause mortality	1.00	1.07 (0.91–1.25)	1.15 (0.96–1.38)	1.08 (1.01–1.16)	1.07 (0.99–1.14)	1.13 (1.02–1.25)	
*n* cases	355	319	226	900	900	900	

Abbreviations: ABSI, a body shape index; BMI, body mass index; CI, confidence interval; HR, hazard ratio; NPCR, National Prostate Cancer Register.

^a^ Prostate cancer risk categories including localised low risk, T1‐2, Gleason score 2–6 and PSA < 10 ng/ml; localised intermediate risk, T1‐2, Gleason score 7 and/or PSA 10 to <20 ng/ml; localised high risk, T3 and/or Gleason score 8–10 and/or PSA 20 to <50 ng/ml; regionally metastatic/locally advanced, T4 and/or N1 and/or PSA 50 to <100 ng/ml in the absence of distant metastases; distant metastases, M1 and/or PSA ≥100 ng/ml.

^b^ Hazard ratios in the full population analyses for waist circumference were calculated using Cox regression with attained age as time scale, stratified on cohort and birth decade (<1935, 1935–1939, 1940–1944, 1945–1949 and ≥1950), and adjusted for age at study enrolment (continuous), height (continuous), smoking status (never smoker, former smoker, current smoker, missing), healthcare region (North of Sweden, South of Sweden), country of birth (born in Sweden with both parents born in Sweden, born in Sweden with one parent born in Sweden, born in Sweden with both parents born abroad, born abroad), highest education (pre‐upper secondary school <9 years, pre‐upper secondary school 9 years, max 2 years upper secondary school, 3 years upper secondary school, post‐upper secondary school <3 years, post‐upper secondary school ≥3 years and university, missing). Hazard ratios of waist circumference were corrected for a regression dilution ratio (RDR) of 0.85 for the full population. Hazard ratios of BMI were corrected for an RDR of 0.90 for the full population.

^c^ *p* values for the heterogeneity in hazard ratios per 5 unit increment between prostate cancer risk categories were calculated using the Lunn and McNeil duplication method.

^d^ Includes additionally 891 prostate cancer cases not categorised into a prostate cancer risk category.

^e^ For the case‐only analyses, categorical adjustments were the same as in the full population analyses for smoking status, healthcare region and country of birth, and additional adjustments were made for age at PCa diagnosis—continuous; highest education closest to diagnosis, income closest to diagnosis—<158, 158–193, 193–230, ≥230 kSEK/year, missing; source of income closest to diagnosis—work, studies, care of child/family, sick, unemployed, early retirement, social benefits, labour market policy activity, pensioner, no income, missing; civil status—unmarried, married, divorced, widower, missing; comorbidity according to the Charlson comorbidity index—none, mild, severe; primary treatment—conservative, curative, non‐curative, missing; and PCa risk category (in the total analysis). Hazard ratios of waist circumference were corrected for a regression dilution ratio (RDR) of 0.91 for cases only. Hazard ratios of BMI were corrected for an RDR of 0.90 for cases only.

^f^ The analysis of BMI was to explore the association in this selected population, using models stratified and adjusted for the same variables as for WC with the exception of height.

ABSI was not associated with the risk of PCa in total or in any cancer risk category. The HR per SD was 0.97; 95% CI 0.93–1.00 for total PCa, 0.97; 95% CI 0.93–1.02 for localised PCa, and 0.96; 95% CI 0.86–1.06 for advanced PCa (Table 3). No associations were found between ABSI and PCa‐specific mortality in the full population (HR per SD, 0.99; 95% CI 0.90–1.09) or among PCa cases only (HR per SD, 0.96; 95% CI 0.85–1.08), nor with all‐cause mortality (HR per SD, 1.07, 95% CI 0.99–1.14) among PCa cases.

We found a negative association between BMI and the risk of localised PCa (HR per 5 kg/m^2^, 0.90; 95% CI 0.85–0.97) and no association with advanced PCa (HR per 5 kg/m^2^, 1.04; 95% CI 0.90–1.21) (Table 3), which are in agreement with our previous study of BMI and PCa risk with a much larger study sample (HRs per 5 kg/m^2^, 0.93; 95% CI 0.91–0.96, and 1.01; 95% CI 0.97–1.06, respectively). The lack of association between BMI and death from PCa in the full population (HR per 5 kg/m^2^, 1.13; 95% CI 0.97–1.35) and among PCa cases (HR per 5 kg/m^2^, 1.17; 95% CI 0.98–1.42) were about the same magnitude as of our previously found associations between BMI and PCa‐specific mortality (HRs per 5 kg/m^2^, 1.12; 95% CI 1.08–1.17, and 1.13; 95% CI 1.08–1.20, respectively).

## 4 DISCUSSION

In this prospective pooled cohort study, we found a negative association for WC with the risk of localised PCa, partially driven by PCa cases detected by asymptomatic PSA‐testing, but no associations with the risk of advanced PCa or PCa‐specific mortality.

Our results for localised PCa are in line with previous studies on WC and PCa risk, which indicated a negative or null association with localised PCa.^3^, ^4^, ^7^, ^9^, ^10^ Several factors have been hypothesised to influence the negative association between WC and localised PCa risk, including an enlarged prostate gland, hemodiluted PSA levels and less active PCa screening among men with obesity.^14^, ^15^, ^16^ Results from our previous study of BMI and PCa risk showed that the negative association with localised PCa was partially driven by the results for asymptomatic PSA‐testing,^5^ as with our negative association between WC and localised PCa that was most evident for asymptomatic PCa cases detected through a PSA‐test. This could indicate that more active health‐seeking and screening behaviour among men with normal weight contributes to their higher risk of localised PCa.

In this study on waist measures, like in our prior study on BMI,^5^ we found no association with the risk of more advanced PCa. Our findings are in agreement with the lacking association between WC, waist–hip ratio and risk of advanced PCa found in the large study by Genkinger et al,^6^ and of a Mendelian randomisation study.^42^ Both were published after the World Cancer Research Fund International's Continuous Update Project report on PCa^43^ and the Umbrella review of the literature on adiposity and cancer at major anatomical sites,^44^ which suggested some evidence of an increased risk of advanced PCa with higher body fatness. These later studies carry large weight in the field, and point at a limited role for waist measures in advanced PCa risk.

Although the association between BMI and PCa‐specific mortality in our and other studies has been consistently positive,^18^, ^19^, ^20^, ^21^, ^22^ results from studies investigating WC and death from PCa have been inconsistent.^3^, ^6^, ^23^, ^24^ Some of the inconsistency could relate to smaller sample sizes in these studies as compared to studies of BMI. For example, a weak positive association between WC and PCa‐specific mortality in the study by Genkinger et al^6^ is of the same magnitude as our non‐significant finding, including in our case‐only analysis, which controls for stage at diagnosis and any potential detection bias that may be at play in a full‐population analysis. The emerging picture of an increased risk of PCa‐specific mortality, but not advanced disease, for obesity assessed by BMI and to lesser extent by WC, appears contradictory. Markers of abdominal obesity such as WC reflect metabolic aberrations more strongly than does BMI and could potentially clarify the relationship between obesity and PCa. However, the findings of our and other studies have not shown clearer or different associations for adiposity markers than for BMI in relation to PCa incidence and mortality.

Strengths of this study include the prospective study design, the population‐based data, the large sample size providing enough statistical power to investigate abdominal adiposity in relation to localised and advanced PCa risk separately, detailed and highly valid PCa data from the NPCR^37^, ^38^ and on confounders, and the availability of repeated measures to correct for short‐ and long‐term intra‐individual variation of WC, which, uncorrected, would result in underestimated hazard ratios.^41^ Limitations of this study concern the smaller sample size and therefore less robust results in the individual cancer risk categories and by mode of PCa detection compared to our larger source population.

In conclusion, the findings of WC in this study support the results from our previous study of BMI in a larger source population of negative associations with the risk of total PCa and localised PCa, partially driven by PSA‐detected PCa cases, and no association with clinically manifest PCa. The emerging picture of an increased risk of PCa‐specific mortality, but not advanced disease, for obesity assessed by BMI and to lesser extent by WC, appears contradictory and requires further examination.

## CONFLICT OF INTEREST

We declare no competing interests.

## AUTHOR CONTRIBUTIONS

Sylvia H J Jochems: Conceptualisation, Data curation, Formal analysis, Writing—original draft and Writing—review and editing. Tanja Stocks: Funding acquisition, Conceptualisation, Writing—original draft and Writing—review and editing. Angela M Wood: Data curation and Writing—review and editing. Christel Häggström: Writing—review and editing. Marju Orho‐Melander: Writing—review and editing. Pär Stattin: Writing—review and editing.

## ETHICS

The study was approved by the Ethics Committee at Lund University, Sweden (No. 2016/564), and informed consent was obtained. This study was performed in accordance with the Declaration of Helsinki.

## Supporting information

Table S1

## Data Availability

The data that support the findings of this study are not publicly available, but data can be made available upon reasonable request.
