# Supplementary material for: Waist circumference and a body shape index and prostate cancer risk and mortality
Source: Cancer Med. 2021 Mar 12;10(8):2885–96. doi: 10.1002/cam4.3827 (PMC8026929; doi:10.1002/cam4.3827)
Supplement: Supplementary file 1 — Table S1 [file CAM4-10-2885-s001.docx]

**Appendix**

Table A1: Baseline characteristics of the 58 457 men in the study according to cohort

| Baseline characteristic | Västerbotten Intervention Programma (VIP) | Northern Sweden Monica Study (MONICA) | Malmö Diet and Cancer Study (MDCS) | Malmö Preventive Project (MPP) |
| --- | --- | --- | --- | --- |
|  | (n= 37 396) | (n= 4000) | (n= 11 615) | (n= 5446) |
| Year of baseline examination | 2003-2016 | 1986-2014 | 1991-1996 | 2002-2006 |
| Total follow-up time, yrs |  |  |  |  |
| Mean (SD) | 8.2 (3.5) | 15.4 (8.8) | 17.3 (7.5) | 9.9 (3.7) |
| Age at study enrolment, yrs |  |  |  |  |
| Mean (SD) | 49.5 (8.4) | 49.7 (14.1) | 58.9 (7.0) | 66.4 (6.7) |
| Waist circumference, cm |  |  |  |  |
| Mean (SD) | 97.2 (11.2) | 94.4 (10.6) | 94.0 (10.2) | 98.3 (10.5) |
| Height, cm |  |  |  |  |
| Mean (SD) | 179.1 (6.6) | 176.7 (7.0) | 176.3 (6.7) | 176.2 (6.6) |
| Weight, kg |  |  |  |  |
| Mean (SD) | 86.8 (14.0) | 83.0 (13.3) | 81.9 (12.3) | 84.8 (13.2) |
| Body mass index, kg/m^2^ |  |  |  |  |
| Mean (SD) | 27.0 (4.0) | 26.6 (3.8) | 26.3 (3.5) | 27.3 (3.9) |
| Smoking status, n (%) |  |  |  |  |
| Never smoker | 25 504 (68) | 1954 (49) | 3135 (27) | 1476 (27) |
| Ex-smoker | 7269 (19) | 1254 (31) | 4621 (40) | 2710 (50) |
| Current smoker | 3948 (11) | 774 (19) | 3030 (26) | 1119 (20) |
| Missing | 675 (2) | 18 (1) | 829 (7) | 141 (3) |
| Highest education, n (%)† |  |  |  |  |
| Pre-upper secondary school <9 yrs | 1536 (4) | 837 (21) | 3361 (29) | 1475 (27) |
| Pre-upper secondary school 9 yrs | 3222 (9) | 265 (7) | 670 (6) | 409 (8) |
| Max. 2 yrs upper secondary school | 14 445 (39) | 1231 (31) | 2858 (24) | 1427 (26) |
| 3 yrs upper secondary school | 5663 (15) | 675 (17) | 2206 (19) | 1019 (19) |
| Post-upper secondary school <3 yrs | 5569 (15) | 453 (11) | 1068 (9) | 478 (8) |
| Post-upper secondary school ≥3 yrs | 6896 (18) | 485 (12) | 1362 (12) | 600 (11) |
| Missing | 65 (<1) | 54 (1) | 90 (1) | 38 (1) |
| Country of birth, n (%) |  |  |  |  |
| Born in Sweden and both parents born in Sweden | 33 496 (90) | 3473 (87) | 9757 (84) | 4390 (81) |
| Other | 3900 (10) | 527 (13) | 1858 (16) | 1056 (19) |

SD=standard deviation.

†Determined by the Swedish Longitudinal integration database for health insurance and labour market studies.
